# Association Between Thyroid Function Indicators and Metabolic‐Associated Fatty Liver Disease: Effect Modification by Iodine Nutritional Status

**DOI:** 10.1155/ije/9291433

**Published:** 2026-06-20

**Authors:** Wei Huang, Jiang Yao, Yunzhen Shi, Heyong Huang

**Affiliations:** ^1^ Department of Surgery, Changxing People’s Hospital, Huzhou, 313100, Zhejiang, China, cxrmyy.com; ^2^ Department of Thyroid and Breast Surgery, The Second Affiliated Hospital of Shandong University of Traditional Chinese Medicine, Jinan, 250001, Shandong, China, bucm.edu.cn; ^3^ EICU, Changxing People’s Hospital, Huzhou, 313100, Zhejiang, China, cxrmyy.com

**Keywords:** effect modification, iodine nutritional status, metabolic-associated fatty liver disease, thyroid function

## Abstract

**Background:**

The relationship between thyroid function indicators and metabolic dysfunction‐associated fatty liver disease (MAFLD) has yielded contradictory results. This study aimed to explore the association between thyroid function indicators and MAFLD, as well as the modifying effect of iodine nutritional status.

**Methods:**

This cross‐sectional analysis utilized nationally representative data from the 2009–2012 National Health and Nutrition Examination Survey (NHANES). Different multivariate logistic regression models were constructed to investigate the association between thyroid function indicators and MAFLD. Mediation analyses were performed to explore the mediating roles of MAFLD diagnosis‐related variables. Lastly, we analyzed the relationship between thyroid function indicators and MAFLD across different iodine nutritional statuses.

**Results:**

After adjusting for covariates unrelated to MAFLD diagnosis, elevated FT3 and FT3/FT4 and decreased FT4 levels increased the odds of MAFLD across three adjusted models (all *p* < 0.05). Besides, FT3 and FT3/FT4 may be associated with MAFLD through potential pathways involving ALT levels, HDL_C levels, or BMI (*p* for indirect effect < 0.05). Moreover, the association between thyroid function and MAFLD may vary depending on iodine nutritional status. Specifically, FT4 exhibited an inverse relationship under iodine‐deficient conditions, whereas elevated FT3 and FT3/FT4 levels were positively associated with MAFLD in iodine‐adequate populations. Under iodine‐excessive conditions, TSH and FT3/FT4 showed significant involvement in MAFLD (*p* < 0.05).

**Conclusion:**

Thyroid function indicators are associated with MAFLD. This association may potentially be mediated by ALT, HDL_C, and BMI and appears to vary depending on iodine nutritional status, though this modifying effect warrants further validation.

## 1. Introduction

Metabolic‐associated fatty liver disease (MAFLD) is a new definition proposed by a panel of international experts from 22 countries in 2020 [1, 2]. MAFLD highlights metabolic abnormalities as the key characteristic in affected individuals [3] and is prevalent globally, with a prevalence of 39.1% [4] in the United States and a combined global rate of 39.22% [5]. From an epidemiological perspective, MAFLD is now a primary contributor to hepatocellular carcinoma and cirrhosis [6]. It also significantly increases the risk of mortality from all causes and cardiovascular diseases [7, 8], especially among patients with diabetes or cardiometabolic conditions [9]. Given the rising global rates of obesity and diabetes, the future trend of the MAFLD epidemic is expected to become more severe [10, 11]. This trend is further aggravated by modern dietary patterns characterized by excessive consumption of energy, carbohydrates, and lipids, alongside deficiencies in polyunsaturated fatty acids, vitamins, and essential minerals [12]. Consequently, the precise identification of individuals with MAFLD is essential for improving their long‐term outcomes [13].

Emerging evidence indicated that fluctuations in thyroid function parameters within the euthyroid range may be linked to the development and progression of MAFLD [14–16]. From a physiological perspective, thyroid hormones exert significant influences on human metabolic processes through multiple mechanisms, particularly in lipid metabolism and energy expenditure [17]. Additionally, imbalances in thyroid hormone levels may lead to various adverse consequences and promote the onset and progression of multiple diseases, including MAFLD [18]. However, the research results on the relationship between thyroid function parameters and MAFLD are inconsistent, ranging from a strong association to no association [19–21].

Additionally, few studies have empirically explored how iodine nutritional status influences the association between thyroid function and MAFLD [22]. Iodine is an essential component of triiodothyronine (T3) and thyroxine (T4), a necessary nutrient for the synthesis of thyroid hormones, and is closely related to the physiological homeostasis of the thyroid hormone cycle [23]. One study conducted in China revealed that the association between urinary iodine concentrations in adults and metabolic disorders and their related diseases is U‐shaped [24]. Iodine may be a potential modifying factor in the association between thyroid function and MAFLD.

Therefore, we conducted a large‐scale cross‐sectional study to evaluate the potential association between each thyroid function indicator and MAFLD and further explore the potential modifying effect of iodine nutritional status. We hypothesized that, even without clinically apparent thyroid disorders, thyroid function is associated with increased odds of MAFLD.

## 2. Methods

### 2.1. Data Source and Study Participants

**FIGURE 1:**
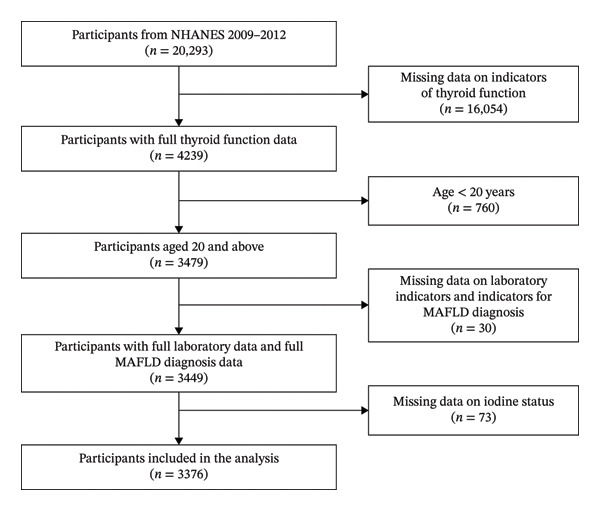
Flowchart of participant selection.

The data used in this study were derived from the National Health and Nutrition Examination Surveys (NHANES), a cross‐sectional population survey designed to estimate the nutritional and health status of both adults and children in the United States. The National Center for Health Statistics’ Research Ethics Review Board and the Centers for Disease Control and Prevention have approved the study, and all participants have provided written informed consent. Based on the availability of information on thyroid function and MAFLD, two cycles of NHANES data collected between 2009 and 2012, comprising a total of 20,293 individuals, were included in this analysis. First, individuals with missing data on thyroid function indicators were excluded (*n* = 16,054). Next, participants younger than 20 years of age were removed (*n* = 760). Subsequently, those lacking complete laboratory data (including metabolic markers) and variables required for MAFLD diagnosis were excluded (*n* = 30). Finally, participants with missing information on iodine status were eliminated (*n* = 30). After excluding participants who did not meet the inclusion criteria, this study ultimately successfully recruited 3376 subjects aged 20 years and above. The detailed flowchart of the inclusion and exclusion criteria for the final participants is illustrated in Figure 1.

### 2.2. MAFLD Diagnosis

A study utilizing NHANES data investigated the agreement between the prevalence of NAFLD assessed by transient elastography and the hepatic steatosis index (HSI), reporting a substantial consistency of 74.27% and an area under the receiver operating characteristic curve (AUROC) of 0.82 for HSI [25]. Therefore, we employed the HSI, a widely validated, economical, and noninvasive measure of steatosis, to define hepatic steatosis [26–28] due to the lack of transient elastography data in NHANES cycles 2009–2012. The detailed calculation formula was as shown below: HSI = 8 × ALT/AST + BMI + 2 (if diabetes) + 2 (if female) [29, 30]. Hepatic steatosis was defined as present in the HSI ≥ 36 [31]. This cutoff value has been previously validated with an accuracy and specificity of 85.9% and 92.4%, respectively [32].

The diagnosis of MAFLD was established based on the presence of hepatic steatosis coupled with at least one of the following three criteria: overweight or obesity (body mass index [BMI] ≥ 25 kg/m^2^), type 2 diabetes mellitus (hemoglobin A1c [HbA1c] ≥ 6.5% or the use of antidiabetic drugs), and metabolic irregularities (at least two metabolic risk abnormalities) [33]. The diagnostic criteria of metabolic abnormalities were as follows [2]: (1) blood pressure ≥ 130/85 mmHg or the use of antihypertensive drugs; (2) waist ≥ 102 cm for males and ≥ 88 cm for females; (3) plasma triglyceride (TG) levels ≥ 150 mg/dL, or plasma high‐density lipoprotein cholesterol (HDL_C) levels < 40 mg/dL for men and < 50 mg/dL for women, or the use of antihyperlipidemic drugs; and (4) prediabetes (i.e., fasting plasma glucose (FPG) within the range of 5.6–6.9 mmol/L, or HbA1c ranging from 5.7% to 6.4%, or 2‐h postload plasma glucose levels (2h‐OGTT) within the range of 7.8–11.0 mmol/L).

### 2.3. Thyroid Function Indicators and Iodine Nutritional Status

This study included nine thyroid function indicators, including free T3 (FT3), free T4 (FT4), FT3/FT4, thyroid‐stimulating hormone (TSH), thyroglobulin antibodies (TgAb), thyroid peroxidase antibodies (TPOAb), thyroglobulin (Tg), TSH index (TSHI), and thyrotropin thyroxine resistance index (TT4RI). TSHI and TT4RI were calculated using the following formulas: TSHI = Log(TSH) + 0.1345 × FT4; TT4RI = FT4 × TSH. The remaining thyroid parameters, including FT3, FT4, TSH, TgAb, TPOAb, and Tg, were measured by the Access 2 method (see NHANES Laboratory Procedures Manual for details, which can be accessed at https://wwwn.cdc.gov/Nchs/Data/Nhanes/Public/2009/DataFiles/THYROD_F.htm.

Iodine concentrations were measured in urine samples by ICP‐DRC‐MS (Inductively Coupled Plasma Dynamic Reaction Cell Mass Spectroscopy). The World Health Organization recommends urinary iodine as the primary biomarker for evaluating iodine nutritional status in epidemiological investigations [34]. A sample size of approximately 500 urine specimens has been shown to provide a sufficiently accurate estimation of population‐level iodine levels [35]. Consequently, this study utilized urinary iodine levels as a biomarker to assess the population’s iodine nutritional status, with a sufficiently large sample size to ensure reliable representation of the target population. Iodine nutritional status was categorized as deficient (< 100 μg/L), adequate (100–299 μg/L), and excessive (≥ 300 μg/L) [36].

### 2.4. Covariates

The research considered many covariates that could potentially influence thyroid function and MAFLD: age, gender (male, female), race (Hispanic, Non‐Hispanic White, Non‐Hispanic Black, and others), marital status (married, others), education level (high school or below, college or above), poverty income ratio (PIR), BMI (kg/m^2^), waist (cm), drinking (yes, no), smoking (never, former, current), fasting plasma glucose (FPG, mg/dL), 2h‐OGTT (mg/dL), hemoglobin A1c (HbA1c, %), systolic blood pressure (SBP, mmHg), diastolic blood pressure (DBP, mmHg), albumin (g/dL), alanine aminotransferase (ALT, U/L), aspartate aminotransferase (AST, U/L), alkaline phosphatase (ALP, IU/L), gamma glutamyl transferase (GGT, U/L), uric acid (mg/dL), platelet count (1000 cells/µL), triglyceride (TG, mg/dL), low‐density lipoprotein cholesterol (LDL_C, mg/dL), HDL_C (mg/dL), and total cholesterol (TC, mg/dL).

### 2.5. Statistical Analysis

Continuous variables were expressed as the median with interquartile range (IQR), while categorical variables were presented as counts with percentages. Differences between groups were evaluated using the Mann–Whitney *U*‐test for continuous variables and the chi‐square test for categorical variables. All statistical analyses were performed using SPSS Version 26.0 and R Version 4.2.1, and *p* values < 0.05 (two‐sided) were considered statistically significant.

Restricted cubic splines (RCS) analysis is a statistical method commonly employed to model nonlinear relationships. It operates by fitting curvilinear relationships using piecewise polynomial functions, while incorporating constraint conditions to prevent overfitting, thereby providing a more accurate representation of the nonlinear associations between independent and dependent variables. Therefore, we fitted the RCS curves to investigate the nonlinear relationship between thyroid function and MAFLD. Collinearity or multicollinearity problems will lead to biased coefficient estimation and a loss of power [37]. Variance inflation factors (VIF) were calculated to evaluate the collinearity of the potential variables, and we did not include variables with a VIF ≥ 3 [38]. Logistic regression examines the relationship between one or several independent variables and a binary dependent variable [39]. Therefore, the relationship between thyroid function indicators and MAFLD was investigated by three distinct multivariate logistic regression models after excluding the variables with collinearity. Model 1 was adjusted for laboratory indicators including GGT, uric acid, albumin, AST, ALT, ALP, TG, LDL_C, HDL_C, platelet, 2h‐OGTT, and HbA1c; Model 2 was adjusted for socio‐demographic characteristics including age, race, education, PIR, drinking, and smoking; Model 3 was adjusted for other variables including BMI, SBP, DBP, and iodine nutritional status. However, excessive adjustment of variables related to MAFLD diagnosis may mask the true effects of the exposure factors. Consequently, we constructed Model 4 (adjusted for GGT, uric acid, albumin, ALP, LDL_C, and platelet) and Model 5 (adjusted for iodine nutritional status) to further examine the association between thyroid function and MAFLD after excluding MAFLD diagnosis‐related variables. According to mediation analysis theory, establishing a mediation effect requires four sequential conditions [40]: First, a statistically significant association must exist between the independent variable and the dependent variable. Second, the independent variable must significantly predict the hypothesized mediator. Third, when both the independent variable and mediator are included in the model, the mediator must retain a significant relationship with the dependent variable. Fourth, the total effect of the independent variable on the outcome must exceed the absolute value of the direct effect observed in the full mediation model, thereby confirming that the indirect effect explains a substantive portion of the total association. Given that MAFLD diagnosis‐related variables may serve as mediators in the relationship between thyroid function and MAFLD, a series of mediation analyses were performed. Additionally, we transformed continuous thyroid function indicators into binary variables using RCS cutoff values to explore their potential associations with MAFLD. Lastly, to examine the potential modification effect of iodine nutritional status, we analyzed the relationship between thyroid function indicators (as binary variables) and MAFLD across different iodine nutritional statuses. E‐value was proposed to assess potential unmeasured or residual confounding in observational studies, and its calculation formula is as follows: OR (odds ratio) = OR + sqrt {OR × (OR‐1)} [41]. Hence, we evaluated the robustness of results by calculating the E‐value. To evaluate the stability of the mediating effect, the indirect effect estimate is transformed into an OR for computational purposes.

## 3. Results

### 3.1. Descriptive Results

This analysis included a total of 3376 adult participants, comprising 1692 women (50.12%) and 1684 men (49.88%), with a median age of 61 years. The minimum sample size calculated by G∗Power software (Version 3.1) was 698 (effect size = 0.3, *α* = 0.05, power = 0.95), so the sample size of this study met the requirement. As shown in Table 1, participants with MAFLD were older, Hispanic, less educated, with lower PIR, and more likely to be nondrinkers and former smokers than those without MAFLD (all *p* < 0.05). We also found a higher BMI, waist, and proportion of adequate iodine nutritional status in MAFLD participants (all *p* < 0.05). Moreover, MAFLD patients exhibited higher levels of FPG, 2h‐OGTT, HbA1c, SBP, DBP, albumin, ALT, AST, ALP, GGT, uric acid, platelet, TG, LDL_C, and TC, along with lower levels of HDL_C (all *p* < 0.05). Intergroup differences were also observed in thyroid function indicators, including FT3, FT4, FT3/FT4, and TSH (all *p* < 0.05). Therefore, the subsequent analysis focused on the relationship between these four thyroid function indicators and MAFLD. There was no statistically significant difference in gender, marital status, TgAb, TPOAb, Tg, TSHI, and TT4RI (all *p* < 0.05).

**TABLE 1:** Comparison of baseline characteristics according to incidence of MAFLD.

Variable	Overall (*n* = 3376)	Non‐MAFLD (*n* = 1527)	MAFLD (*n* = 1849)	*p*‐value
Age, years	48.00 (34.00, 63.00)	46.00 (30.00, 64.00)	49.00 (37.00, 62.00)	0.001
Gender, *n* (%)				0.050
Male	1684 (49.88)	790 (51.73)	894 (48.35)	
Female	1692 (50.12)	737 (48.27)	955 (51.65)	
Race, *n* (%)				< 0.001
Hispanic	834 (24.70)	288 (18.86)	546 (29.53)	
Non‐Hispanic White	1443 (42.74)	706 (46.23)	737 (39.86)	
Non‐Hispanic Black	706 (20.91)	283 (18.53)	423 (22.88)	
Others	393 (11.64)	250 (16.37)	143 (7.73)	
Marital status, *n* (%)				0.097
Married	1724 (51.08)	756 (49.51)	968 (52.38)	
Others	1651 (48.92)	771 (50.49)	880 (47.62)	
Education, *n* (%)				< 0.001
High school or below	1598 (47.38)	653 (42.82)	945 (51.14)	
College or above	1775 (52.62)	872 (57.18)	903 (48.86)	
PIR	2.010 (1.07, 3.98)	2.180 (1.09, 4.14)	1.86 (1.05, 3.80)	0.018
BMI, kg/m^2^	27.72 (24.13, 32.40)	23.81 (21.90, 25.70)	31.80 (29.00, 35.96)	< 0.001
Waist, cm	97.10 (87.00, 108.70)	86.30 (80.20, 93.10)	107.00 (98.80, 116.10)	< 0.001
Drinking, *n* (%)				0.006
Yes	2236 (73.50)	1036 (75.95)	1200 (71.51)	
No	806 (26.50)	328 (24.05)	478 (28.49)	
Smoking, *n* (%)				0.009
Never	1881 (55.73)	853 (55.86)	1028 (55.63)	
Former	819 (24.27)	340 (22.27)	479 (25.92)	
Current	675 (20.00)	334 (21.87)	341 (18.45)	
Iodine nutritional status, *n* (%)				0.002
Deficient	1191 (35.28)	585 (38.31)	606 (32.77)	
Adequate	1614 (47.81)	683 (44.73)	931 (50.35)	
Excessive	571 (16.91)	259 (16.96)	312 (16.87)	
FPG (mg/dL)	100.00 (93.00, 110.00)	96.00 (90.00, 104.00)	103.00 (95.00, 115.00)	< 0.001
2h‐OGTT (mg/dL)	108.00 (88.00, 135.00)	101.00 (83.00, 127.00)	116.00 (95.00, 145.00)	< 0.001
HbA1c (%)	5.50 (5.20, 5.90)	5.40 (5.20, 5.70)	5.70 (5.30, 6.20)	< 0.001
SBP (mmHg)	120.00 (110.00, 134.00)	118.00 (108.00, 132.00)	122.00 (114.00, 134.00)	< 0.001
DBP (mmHg)	70.00 (62.00, 78.00)	70.00 (62.00, 76.00)	72.00 (64.00, 80.00)	< 0.001
Albumin (g/dL)	4.30 (4.10, 4.50)	4.40 (4.10, 4.60)	4.20 (4.00, 4.40)	< 0.001
ALT (U/L)	21.00 (16.00, 28.00)	18.00 (15.00, 23.00)	23.00 (18.00, 33.00)	< 0.001
AST (U/L)	23.00 (20.00, 28.00)	23.00 (20.00, 27.00)	24.00 (20.00, 29.00)	0.001
ALP (U/L)	64.00 (53.00, 79.00)	61.00 (50.00, 75.00)	67.00 (55.00, 81.00)	< 0.001
GGT (U/L)	19.00 (14.00, 30.00)	16.00 (12.00, 23.00)	23.00 (16.00, 35.00)	< 0.001
Uric acid (mg/dL)	5.30 (4.40, 6.40)	4.90 (4.10, 5.90)	5.70 (4.70, 6.70)	< 0.001
Platelet (1000 cells/µL)	230.00 (196.00, 270.00)	224.00 (191.00, 265.00)	235.00 (201.00, 274.00)	< 0.001
TG (mg/dL)	105.00 (75.00, 151.00)	88.00 (63.00, 124.00)	121.00 (86.00, 173.00)	< 0.001
LDL_C (mg/dL)	114.00 (91.00, 139.00)	109.00 (89.00, 134.00)	118.00 (93.00, 142.00)	< 0.001
HDL_C (mg/dL)	50.00 (42.00, 61.00)	56.00 (45.00, 67.00)	46.00 (39.00, 55.00)	< 0.001
TC (mg/dL)	192.00 (165.00, 220.00)	188.00 (162.00, 215.00)	194.00 (167.00, 223.00)	< 0.001
FT3 (pg/mL)	3.13 (2.90, 3.40)	3.10 (2.89, 3.35)	3.17 (2.92, 3.40)	< 0.001
FT4 (pmol/L)	10.30 (9.30, 11.60)	10.40 (9.40, 11.60)	10.30 (9.00, 11.60)	< 0.001
FT3/FT4	0.30 (0.27, 0.34)	0.30 (0.26, 0.34)	0.31 (0.27, 0.35)	< 0.001
TSH (mIU/L)	1.50 (1.02, 2.21)	1.45 (0.98, 2.18)	1.53 (1.06, 2.23)	0.028
TgAb (IU/mL)	0.60 (0.60, 0.60)	0.60 (0.60, 0.60)	0.60 (0.60, 0.60)	0.223
TPOAb (IU/mL)	0.60 (0.30, 1.60)	0.60 (0.30, 1.60)	0.60 (0.30, 1.70)	0.265
Tg (ng/mL)	10.04 (5.66, 17.22)	9.69 (5.65, 16.91)	10.49 (5.68, 17.53)	0.191
TSHI	1.59 (1.38, 1.81)	1.59 (1.37, 1.82)	1.58 (1.38, 1.79)	0.316
TT4RI	15.49 (10.67, 23.22)	15.21 (10.19, 23.44)	15.67 (11.09, 22.93)	0.263

*Note:* Median (IQR) for continuous variables and counts (percentage) for categorical variables. MAFLD, metabolic dysfunction‐associated fatty liver disease; 2h‐OGTT, 2‐h postload plasma glucose; HbA1c, hemoglobin A1c; ALT, alanine aminotransferase; AST, aspartate aminotransferase; ALP, alkaline phosphatase; TG, triglyceride; FT3, free triiodothyronine; FT4, free thyroxine; TgAb, thyroglobulin antibodies; TPOAb, thyroid peroxidase antibodies; Tg, thyroglobulin; TSHI, TSH index; TT4RI, thyrotropin thyroxine resistance index.

Abbreviations: BMI, body mass index; DBP, diastolic blood pressure; FPG, fasting plasma glucose; GGT, gamma glutamyl transferase; HDL_C, high‐density lipoprotein cholesterol; LDL_C, low‐density lipoprotein cholesterol; PIR, poverty income ratio; SBP, systolic blood pressure; TC, total cholesterol; TSH, thyroid‐stimulating hormone.

### 3.2. Association Between Thyroid Function Indicators on a Continuous Scale and MAFLD

**FIGURE 2:**
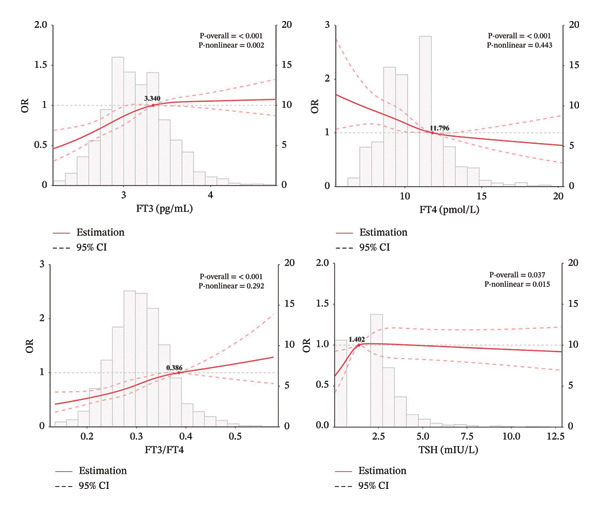
Restricted cubic splines for dose–response associations between indicators of thyroid function and MAFLD. The red dotted lines represent the 95% confidence interval. The cutoff values for FT3, FT4, FT3/FT4, and TSH were 3.340 pg/mL, 11.796 pmol/L, 0.396, and 1.402 mIU/L, respectively. Abbreviations: MAFLD, metabolic dysfunction‐associated fatty liver disease; OR, odds ratio; FT3, free triiodothyronine; FT4, free thyroxine; TSH, thyroid‐stimulating hormone.

To investigate the nonlinear relationship between thyroid function and MAFLD, we first fitted RCS curves. Figure 2 indicated a nonlinear association between FT3 and TSH in relation to MAFLD (*p* for nonlinear < 0.05), whereas the relationships involving FT4 and the FT3/FT4 with MAFLD were linear (*p* for nonlinear > 0.05). The curves of FT3 and FT3/FT4 exhibited a general upward trend, whereas the curve of FT4 displayed a clear downward trend (Figure 2). The curve of TSH rose rapidly at low levels and then decreased slightly at higher concentrations (Figure 2). The cutoff values for FT3, FT4, FT3/FT4, and TSH were 3.340 pg/mL, 11.796 pmol/L, 0.396, and 1.402 mIU/L, respectively. After eliminating the variables with collinearity (Table S1), we established three different logistic regression models to explore the association between thyroid function indicators and MAFLD (Table 2). In Model 1, no significant associations were observed, and the ORs for FT3, FT4, FT3/FT4, and TSH were 1.177 (95% confidence interval [CI]: 0.990, 1.651), 0.952 (95% CI: 0.882, 1.028), 6.720 (95% CI: 1.325, 63.401), and 1.005 (95% CI: 0.966, 1.047), respectively. In Model 2, FT3, FT4, and FT3/FT4 were significantly associated with MAFLD, with ORs of 1.566 (95% CI: 1.260, 1.955), 0.937 (95% CI: 0.901, 0.973), and 32.153 (95% CI: 8.787, 120.101), respectively. In Model 3, FT3 and FT3/FT4 were significantly associated with MAFLD, with ORs of 1.771 (95% CI: 1.276, 2.485) and 14.398 (95% CI: 2.075, 121.054). However, FT4 and TSH were not significantly associated with MAFLD, with an OR of 0.970 (95% CI: 0.903, 1.039) and 0.988 (95% CI: 0.950, 1.034).

**TABLE 2:** Association between thyroid function and MAFLD.

Model	Indicators (continuous)	OR (95% CI)	*p*‐value
Model 1	FT3	1.177 (0.990, 1.651)	0.188
	FT4	0.952 (0.882, 1.028)	0.205
	FT3/FT4	6.720 (1.325, 63.401)	0.065
	TSH	1.005 (0.966, 1.047)	0.794

Model 2	FT3	**1.566 (1.260, 1.955)**	**< 0.001**
	FT4	**0.937 (0.901, 0.973)**	**0.001**
	FT3/FT4	**32.153 (8.787, 120.101)**	**< 0.001**
	TSH	0.996 (0.972, 1.019)	0.702

Model 3	FT3	**1.771 (1.276, 2.485)**	**0.001**
	FT4	0.970 (0.903, 1.039)	0.392
	FT3/FT4	**14.398 (2.075, 121.054)**	**0.013**
	TSH	0.988 (0.950, 1.034)	0.549

*Note:* Model 1: adjusted for GGT, uric acid, albumin, AST, ALT, ALP, TG, LDL_C, HDL_C, platelet, 2h‐OGTT, and HbA1c. Model 2: adjusted for age, race, education, PIR, drinking, and smoking. Model 3: adjusted for BMI, SBP, DBP, and iodine nutritional status. MAFLD, metabolic dysfunction‐associated fatty liver disease; FT3, free triiodothyronine; FT4, free thyroxine; ALT, alanine aminotransferase; AST, aspartate aminotransferase; ALP, alkaline phosphatase; TG, triglyceride; 2h‐OGTT, 2‐h postload plasma glucose; HbA1c, hemoglobin A1c. The bold values indicate *p* < 0.05.

Abbreviations: BMI, body mass index; CI, confidence interval; DBP, diastolic blood pressure; GGT, gamma glutamyl transferase; HDL_C, high‐density lipoprotein cholesterol; LDL_C, low‐density lipoprotein cholesterol; OR, odds ratio; PIR, poverty income ratio; SBP, systolic blood pressure; TSH, thyroid‐stimulating hormone.

### 3.3. Association Between Thyroid Function Indicators and MAFLD After Excluding the Variables for Diagnosing MAFLD and the Mediating Effects of the Variables

In Models 1 and 3, several thyroid function indicators were not significantly associated with the outcome. To identify the possible reasons, we conducted the following analyses. First, there was multicollinearity between the variables, which increased the standard error of the coefficients and reduced the statistical power. However, we had already conducted the multicollinearity analysis earlier, and the covariates included in our analysis exhibited no multicollinearity.

Second, over‐adjusting MAFLD diagnosis‐related variables may mask the true effect of the exposure factors. Therefore, after excluding these variables, the relationship between thyroid function indicators and MAFLD was further analyzed. In Models 4 (adjusted for GGT, uric acid, albumin, ALP, LDL_C, and platelet) and Model 5 (adjusted for iodine nutritional status), FT3, FT4 and FT3/FT4 were all significantly associated with MAFLD except for TSH. The ORs in Model 4 for FT3, FT4, FT3/FT4, and TSH were 1.614 (95% CI: 1.214, 2.163), 0.934 (95% CI: 0.883, 0.987), 30.559 (95% CI: 5.415, 184.766), and 0.988 (95% CI: 0.946, 1.022); the ORs in Model 5 were 1.406 (95% CI: 1.179, 1.682), 0.936 (95% CI: 0.904, 0.968), 24.503 (95% CI: 8.106, 75.100), and 0.999 (95% CI: 0.977, 1.023) (Table 3).

**TABLE 3:** Association between thyroid function and MAFLD after excluding the variables for diagnosing MAFLD.

Model	Indicators (continuous)	OR (95% CI)	*p*‐value
Model 4	**FT3**	**1.614 (1.214, 2.163)**	**0.001**
	**FT4**	**0.934 (0.883, 0.987)**	**0.017**
	**FT3/FT4**	**30.559 (5.415, 184.766)**	**< 0.001**
	TSH	0.988 (0.946, 1.022)	0.491

Model 5	**FT3**	**1.406 (1.179, 1.682)**	**< 0.001**
	**FT4**	**0.936 (0.904, 0.968)**	**< 0.001**
	**FT3/FT4**	**24.503 (8.106, 75.100)**	**< 0.001**
	TSH	0.999 (0.977, 1.023)	0.946

*Note:* Model 4: adjusted for GGT, uric acid, albumin, ALP, LDL_C, and platelet. Model 5: adjusted for iodine nutritional status. MAFLD, metabolic dysfunction‐associated fatty liver disease; FT3, free triiodothyronine; FT4, free thyroxine; ALP, alkaline phosphatase. The bold values indicate *p* < 0.05.

Abbreviations: CI, confidence interval; GGT, gamma glutamyl transferase; LDL_C, low‐density lipoprotein cholesterol; OR, odds ratio; TSH, thyroid‐stimulating hormone.

Moreover, we assessed the robustness of the results through E‐value analysis, and the results confirmed the robustness of our main findings (E‐values for FT3, FT4, and FT3/FT4 in Model 4 were 2.61, 1.35, and 60.61, respectively; E‐values for FT3, FT4, and FT3/FT4 in Model 5 were 2.16, 1.34, and 48.50, respectively).

Third, MAFLD diagnosis‐related variables may serve as the mediators between thyroid function indicators and MAFLD. A series of mediation analyses were conducted (Table 4). In mediation Model 1, ALT and HDL_C exerted a complete mediating effect in the relationship between FT3 and MAFLD (ALT: *β* = 0.012, *p* < 0.05; HDL_C: *β* = 0.015, *p* < 0.05), whereas they also played a partial mediating role in the association between FT3/FT4 and MAFLD (ALT: *β* = 0.137, *p* < 0.05; HDL_C: *β* = 0.101, *p* < 0.05). FT4 directly exhibited a direct association with MAFLD (*β* = −0.018, *p* < 0.05), while TSH was not related to MAFLD with a total effect of −0.002 (*p* > 0.05). In mediation Model 2, BMI demonstrated partial mediation effects in both the FT3‐MAFLD (*β* = 0.018, *p* < 0.05) and FT3/FT4‐MAFLD pathways (*β* = 0.235, *p* < 0.05). FT4 exhibited a direct effect on MAFLD (*β* = −0.010, *p* < 0.05), while no significant association was observed between TSH and MAFLD (*β* = −0.001, *p* > 0.05). The mediation analysis findings suggested that FT3 and FT3/FT4 may be linked to MAFLD through potential pathways involving ALT levels, HDL_C levels, or BMI.

**TABLE 4:** The mediating effects of the relationship between thyroid function and MAFLD.

Mediation model		*X* = FT3		*X* = FT4		*X* = FT3/FT4		*X* = TSH	
		*β* (95% CI)	*p*‐value	*β* (95% CI)	*p*‐value	*β* (95% CI)	*p*‐value	*β* (95% CI)	*p*‐value
Model 1	Total effect	**0.041 (0.008, 0.075)**	**0.016**	**−0.019 (**−**0.034,** −**0.004)**	**0.013**	**0.520 (0.229, 0.812)**	**< 0.001**	−0.002 (−0.010, 0.006)	0.612
	Direct effect	0.015 (−0.016, 0.045)	0.345	**−0.018 (**−**0.032,** −**0.005)**	**0.007**	**0.331 (0.066, 0.597)**	**0.014**	0.000 (−0.007, 0.007)	0.964
	X ⟶ ALT ⟶ MAFLD	**0.012 (0.001, 0.095)**	**0.004**	−0.005 (−0.011, 0.000)	0.060	**0.137 (0.024, 0.474)**	**< 0.001**	−0.002 (−0.005, 0.000)	0.060
	X ⟶ AST ⟶ MAFLD	0.001 (−0.015, 0.008)	0.720	0.002 (−0.002, 0.006)	0.344	−0.011 (−0.119, 0.024)	0.604	0.000 (−0.002, 0.001)	0.440
	X ⟶ 2h‐OGTT ⟶ MAFLD	0.000 (−0.008, 0.005)	0.904	0.001 (0.000, 0.005)	0.148	−0.008 (−0.066, 0.015)	0.428	0.000 (−0.001, 0.001)	0.964
	X ⟶ HbA1c ⟶ MAFLD	−0.001 (−0.008, 0.004)	0.268	0.001 (0.000, 0.003)	0.304	−0.018 (−0.061, 0.005)	0.080	0.000 (0.000, 0.002)	0.452
	X ⟶ TG⟶ MAFLD	0.000 (−0.008, 0.002)	0.732	0.001 (−0.001, 0.003)	0.404	−0.012 (−0.090, 0.032)	0.528	0.000 (−0.001, 0.000)	0.660
	X ⟶ HDL_C ⟶ MAFLD	**0.015 (0.006, 0.081)**	**< 0.001**	0.000 (−0.003, 0.004)	0.836	**0.101 (0.043, 0.249)**	**< 0.001**	−0.001 (−0.003, 0.001)	0.252

Model 2	Total effect	**0.046 (0.017, 0.075)**	**0.002**	**−0.016 (**−**0.024,** −**0.007)**	**< 0.001**	**0.566 (0.340, 0.791)**	**< 0.001**	−0.001 (−0.007, 0.005)	0.697
	Direct effect	**0.029 (0.008, 0.049)**	**0.007**	**−0.010 (**−**0.016,** −**0.004)**	**0.001**	**0.339 (0.177, 0.502)**	**< 0.001**	−0.002 (−0.006, 0.002)	0.406
	X ⟶ BMI ⟶ MAFLD	**0.018 (0.001, 0.066)**	**0.004**	−0.006 (−0.012, 0.000)	0.056	**0.235 (0.070, 0.531)**	**0.004**	0.001 (−0.003, 0.009)	0.788
	X ⟶ SBP ⟶ MAFLD	−0.001 (−0.003, 0.001)	0.200	0.000 (0.000, 0.001)	0.168	−0.007 (−0.025, 0.000)	0.112	0.000 (0.000, 0.000)	0.364
	X ⟶ DBP ⟶ MAFLD	0.000 (−0.003, 0.003)	0.984	0.000 (0.000, 0.001)	0.980	0.000 (0.000, 0.001)	0.980	0.000 (0.000, 0.000)	0.784

*Note:* MAFLD, metabolic dysfunction‐associated fatty liver disease; FT3, free triiodothyronine; FT4, free thyroxine; ALT, alanine aminotransferase; AST, aspartate aminotransferase; 2h‐OGTT, 2‐h postload plasma glucose; HbA1c, hemoglobin A1c; TG, triglyceride. The bold values indicate *p* < 0.05.

Abbreviations: BMI, body mass index; CI, confidence interval; DBP, diastolic blood pressure; HDL_C, high‐density lipoprotein cholesterol; SBP, systolic blood pressure; TSH, thyroid‐stimulating hormone.

To assess the robustness of the observed mediating effects, we calculated the E‐value, an indicator used to evaluate the influence of unmeasured confounding factors. The E‐values for ALT and BMI in the FT3/FT4‐MAFLD pathway were 1.52 and 1.78, respectively, indicating that the mediating effects of ALT and BMI in this pathway remain relatively stable. The remaining mediation effects exhibited E‐values ranging from 1.1 to 1.4.

### 3.4. Association Between Thyroid Function Indicators on a Categorical Scale and MAFLD

Based on the aforementioned analyses, we dichotomized continuous thyroid function parameters using RCS‐derived cutoff values and subsequently explored their potential associations with MAFLD in three logistic regression models that excluded MAFLD diagnosis‐related covariates (Table 5). All thyroid function parameters demonstrated statistically significant associations with MAFLD except for TSH in Model 1. In Model 1, the ORs for FT3, FT4, FT3/FT4, and TSH were 1.509 (95% CI: 1.194, 1.911), 0.741 (95% CI: 0.569, 0.965), 1.523 (95% CI: 1.068, 2.188), and 0.869 (95% CI: 0.698, 1.08), respectively. In Model 2, the ORs for FT3, FT4, FT3/FT4, and TSH were 1.388 (95% CI: 1.159, 1.666), 0.746 (95% CI: 0.615, 0.905), 1.562 (95% CI: 1.205, 2.037), and 1.202 (95% CI: 1.029, 1.405). In Model 3, the ORs for FT3, FT4, FT3/FT4, and TSH were 1.315 (95% CI: 1.129, 1.532), 0.757 (95% CI: 0.638, 0.897), 1.643 (95% CI: 1.298, 2.091), and 1.193 (95% CI: 1.041, 1.367).

**TABLE 5:** Association between thyroid function and MAFLD after transforming thyroid function indicators into binary variables based on cutoff values.

Model	Indicators (categorical)	OR (95% CI)	*p*‐value
Model 1	FT3	**1.509 (1.194, 1.911)**	**0.001**
	FT4	**0.741 (0.569, 0.965)**	**0.026**
	FT3/FT4	**1.523 (1.068, 2.188)**	**0.021**
	TSH	0.869 (0.698, 1.08)	0.205

Model 2	FT3	**1.388 (1.159, 1.666)**	**< 0.001**
	FT4	**0.746 (0.615, 0.905)**	**0.003**
	FT3/FT4	**1.562 (1.205, 2.037)**	**0.001**
	TSH	**1.202 (1.029, 1.405)**	**0.020**

Model 3	FT3	**1.315 (1.129, 1.532)**	**< 0.001**
	FT4	**0.757 (0.638, 0.897)**	**0.001**
	FT3/FT4	**1.643 (1.298, 2.091)**	**< 0.001**
	TSH	**1.193 (1.041, 1.367)**	**0.011**

*Note:* Model 1: adjusted for GGT, uric acid, albumin, ALP, LDL_C, and platelet. Model 2: adjusted for age, race, education, PIR, drinking, and smoking. Model 3: adjusted for iodine nutritional status. MAFLD, metabolic dysfunction‐associated fatty liver disease; FT3, free triiodothyronine; FT4, free thyroxine; ALP, alkaline phosphatase. The bold values indicate *p* < 0.05.

Abbreviations: CI, confidence interval; GGT, gamma glutamyl transferase; LDL_C, low‐density lipoprotein cholesterol; OR, odds ratio; PIR, poverty income ratio; TSH, thyroid‐stimulating hormone.

### 3.5. Effect Modification of Iodine Nutritional Status

To examine the potential modification effect of iodine nutritional status, we analyzed the relationship between thyroid function indicators (as binary variables) and MAFLD across different iodine nutritional statuses (Table 6). Under iodine‐deficient conditions, a significant inverse association between FT4 and MAFLD was observed in Model 1 (OR: 0.604, 95% CI: 0.387–0.939). In iodine‐adequate populations, significant associations of both FT3 (OR: 1.582, 95% CI: 1.125–2.233) and FT3/FT4 (OR: 1.995, 95% CI: 1.183–3.445) with MAFLD were observed in Model 1, which persisted in Model 2 (OR: 1.376, 95% CI: 1.054–1.801; OR: 1.732, 95% CI: 1.172–2.601). Notably, under iodine‐excessive conditions, FT3 demonstrated MAFLD association in Model 1 (OR: 1.919, 95% CI: 1.011–3.704), whereas both FT3/FT4 (OR: 2.112, 95% CI: 1.153–4.039) and TSH (OR: 1.776, 95% CI: 1.205–2.632) exhibited significant associations in Model 2.

**TABLE 6:** Association between thyroid function and MAFLD at different iodine nutritional status.

Iodine nutritional status	Indicators (categorical)	Model 1		Model 2	
		OR (95% CI)	*p*‐value	OR (95% CI)	*p*‐value
Deficient	FT3	1.313 (0.898, 1.924)	0.161	1.309 (0.975, 1.761)	0.074
	FT4	**0.604 (0.387, 0.939)**	**0.026**	0.724 (0.518, 1.01)	0.058
	FT3/FT4	1.180 (0.664, 2.128)	0.575	1.059 (0.678, 1.659)	0.802
	TSH	0.972 (0.677, 1.395)	0.878	1.017 (0.783, 1.32)	0.899

Adequate	FT3	**1.582 (1.125, 2.233)**	**0.009**	**1.376 (1.054, 1.801)**	**0.020**
	FT4	0.820 (0.561, 1.198)	0.303	0.782 (0.590, 1.039)	0.089
	FT3/FT4	**1.995 (1.183, 3.445)**	**0.011**	**1.732 (1.172, 2.601)**	**0.007**
	TSH	0.770 (0.561, 1.054)	0.104	1.160 (0.921, 1.462)	0.207

Excessive	FT3	**1.919 (1.011, 3.704)**	**0.048**	1.555 (0.963, 2.537)	0.073
	FT4	0.868 (0.429, 1.745)	0.690	0.672 (0.42, 1.073)	0.096
	FT3/FT4	1.174 (0.458, 3.069)	0.738	**2.112 (1.153, 4.039)**	**0.019**
	TSH	0.989 (0.559, 1.751)	0.969	**1.776 (1.205, 2.632)**	**0.004**

*Note:* Model 1: adjusted for GGT, uric acid, albumin, ALP, LDL_C, and platelet. Model 2: adjusted for age, race, education, PIR, drinking, and smoking. MAFLD, metabolic dysfunction‐associated fatty liver disease; FT3, free triiodothyronine; FT4, free thyroxine; ALP, alkaline phosphatase. The bold values indicate *p* < 0.05.

Abbreviations: CI, confidence interval; GGT, gamma glutamyl transferase; LDL_C, low‐density lipoprotein cholesterol; OR, odds ratio; PIR, poverty income ratio; TSH, thyroid‐stimulating hormone.

## 4. Discussion

This study found that the thyroid function indicators FT3, FT4, and FT3/FT4 were significantly associated with MAFLD after excluding MAFLD diagnosis‐related variables. Besides, FT3 and FT3/FT4 may be related to MAFLD through potential links with ALT levels, HDL_C levels, or BMI. After dichotomizing thyroid function indicators, almost all thyroid function parameters, except for TSH in Model 1, showed statistically significant associations with MAFLD. Moreover, the association between thyroid function indicators and MAFLD may vary depending on iodine nutritional status.

First, thyroid function indicators FT3, FT4, and FT3/FT4 (whether on a continuous scale or a categorical scale) were significantly associated with MAFLD. Consistent with the results of this study, a retrospective cross‐sectional study in China found that the risk of MAFLD was positively associated with the levels of FT3 but negatively associated with FT4 [16]. Another cross‐sectional study in China also indicated that the high‐level FT3/FT4 remained a risk factor for MAFLD even after adjusting for confounders [42]. The mechanism of this association may involve multiple pathways. First, FT3 stimulates lipolysis in adipose tissue, thereby increasing the availability of free fatty acids, which subsequently serve as substrates for hepatic triglyceride synthesis [43]. Moreover, FT3 also promotes de novo lipogenesis in the liver [44]. Second, insulin resistance is intensified by FT3‐induced suppression of insulin receptor substrate phosphorylation and PI3K/Akt signaling pathways, leading to diminished peripheral glucose uptake and increased hepatic gluconeogenesis [45, 46]. Third, mitochondrial dysfunction and oxidative stress arise from FT3‐driven reactive oxygen species (ROS) overproduction and FT4‐associated declines in antioxidant enzymes, including superoxide dismutase (SOD) and glutathione peroxidase (GPx), promoting lipid peroxidation and inflammation through Kupffer cell activation and subsequent release of tumor necrosis factor‐alpha (TNF‐α) and interleukin‐6 (IL‐6) [47, 48]. Fourth, altered peripheral deiodinase (DIO) activity characterized by DIO1/DIO2 upregulation elevates local T3 levels, establishing a T3‐dominant microenvironment that accelerates lipogenesis and inflammation, particularly in adipose and hepatic tissues [49]. These mechanisms synergistically promote hepatic steatosis, insulin resistance, and oxidative injury, establishing a vicious cycle that perpetuates MAFLD progression.

Interestingly, TSH was not significantly associated with MAFLD on a continuous scale in any of the adjustment models but was associated with MAFLD on a categorical scale in Models 2 and 3. Upon examining the RCS curve graph, it becomes evident that the trend of TSH changes exhibited a markedly opposite pattern before and after the cutoff point. Therefore, this situation might be caused by the nonlinear association between TSH and MAFLD. On the one hand, the categorical models preserve nonlinearity but sacrifice granularity, potentially inflating effect sizes. On the other hand, lower TSH levels may enhance hepatic lipogenesis via β‐adrenergic signaling and insulin resistance [46], while higher TSH levels promote dyslipidemia and systemic inflammation [50]. The RCS curve reflects this pattern, where opposing effects mask linear associations in continuous models.

Additionally, ALT, HDL‐C, and BMI were identified as potential mediators in the relationship between thyroid function and MAFLD. Similarly, Fan et al. reported that the association between thyroid hormones and MAFLD is mediated by obesity and metabolic disorders [51]. The mediating effect may be explained by several pathways. First, thyroid hormones promote hepatic fat accumulation; lipotoxicity induces hepatocyte injury and ALT release into the bloodstream [52]; elevated ALT further activates hepatic stellate cells (HSCs), promoting inflammation and fibrosis [53], and thereby driving MAFLD progression. Second, thyroid hormones influence mitochondrial function, thereby impairing the antioxidant and anti‐inflammatory properties of HDL_C and aggravating the pathological progression of MAFLD [47, 54]. Third, thyroid hormones enhance hypothalamic‐sympathetic nervous system activity, promoting white adipose tissue lipolysis and increasing free fatty acid flux to the liver [55]. Additionally, they suppress adipocyte differentiation, resulting in ectopic fat deposition (e.g., liver and muscles). Obesity‐induced adipokine imbalance worsens insulin resistance and hepatic lipid accumulation.

This study also revealed novel insights. The relationship between thyroid function indicators and MAFLD was modified by iodine nutritional status. Iodine nutritional status may dynamically modify the metabolic associations of thyroid markers (FT3, FT4, TSH) by regulating thyroid hormone synthesis, TSH secretion, and DIO activity, leading to differential MAFLD odds [56–58]. Iodine functions both as a pro‐oxidant and an antioxidant, playing a key role in maintaining oxidative homeostasis at the cellular, physiological, and molecular levels [59]. Consequently, iodine deficiency can impair thyroid function, leading to a reduced metabolic rate and an increased risk of metabolic syndrome (MetS) and dyslipidemia [60]. Similarly, excessive iodine intake stimulates increased production of thyroid hormones, which enhances metabolic activity and, in turn, elevates the risk of MetS and dyslipidemia [60]. Dairy products, grains, eggs, and fish are the primary dietary sources of iodine in the typical American diet [61]. Growing evidence suggests that Western dietary patterns, defined by high intakes of saturated fats, refined carbohydrates, and processed meats [62], act as independent contributors to the progression of MAFLD [63–65], primarily through disturbances in lipid metabolism, hepatic inflammation, and insulin resistance. Importantly, these dietary habits may interact with suboptimal iodine status to further impair thyroid function. For example, excessive sugar consumption has been shown to inhibit DIO activity [66], thereby potentially intensifying the effects of iodine deficiency on hypothyroidism. Similarly, diets high in omega‐6 fatty acids may enhance iodine‐related oxidative stress in hepatocytes [67]. In contrast, Mediterranean‐style diets (rich in fiber, seafood, and plant‐derived polyphenols) may offer dual protective benefits by both reducing MAFLD risk [68] and improving iodine bioavailability. Future studies should investigate how specific macronutrient compositions (e.g., low‐carbohydrate vs. low‐fat diets) influence the thyroid‐MAFLD axis across varying levels of iodine nutrition, particularly considering that high‐glycemic‐load foods may directly interfere with thyroid hormone synthesis. These findings underscore the importance of iodine as a key element within a broader dietary framework that collectively influences the development and progression of MAFLD.

Clinically, this study recommends that thyroid function, particularly the FT3/FT4 ratio, be routinely assessed in patients with obesity, diabetes, or MetS as a robust predictor for identifying high‐risk groups of MAFLD. Additionally, personalized early warning indicators can be established by integrating RCS curves. Lastly, clinicians should choose suitable thyroid function indicators according to varying iodine nutritional statuses to enhance the accuracy of MAFLD prediction.

This study also had several limitations. Firstly, the cross‐sectional study design limited the causal inference. Future research should validate these findings through prospective cohort studies. Secondly, the study population comprised American adults, which may limit the generalizability of the findings to other populations. Moreover, the diagnosis of hepatic steatosis in this study relied on the HSI rather than direct imaging modalities (e.g., ultrasound or transient elastography) or histological confirmation. While HSI is a well‐validated and widely adopted noninvasive surrogate in large epidemiological studies, it is inherently imperfect and inevitably introduces the possibility of outcome misclassification bias. More critically, the calculation of HSI heavily incorporates anthropometric and metabolic variables, including BMI, ALT, AST, and diabetes status. This introduces a degree of mathematical coupling, as these variables inherently overlap with the diagnostic criteria for MAFLD and the mediators under investigation. Consequently, our findings—particularly the identification of BMI and ALT as potential mediators in the thyroid‐MAFLD pathway—must be interpreted with high caution, as the outcome variable (MAFLD) was mathematically derived partially from these exact mediating variables. Future prospective studies utilizing gold‐standard imaging or biopsy to define steatosis are imperative to validate these associations and strictly disentangle the biological pathways from algorithmic mathematical overlaps. Fourthly, although NHANES provides data related to dietary quality, it lacks information on dietary iodine, and our study focuses on the modifying effect of iodine nutritional status. Introducing dietary quality might mask the modifying effect of iodine. Crucially, we quantified the impact of potential unmeasured confounding through E‐value analysis, and the results confirmed the robustness of our main findings (E‐values for FT3, FT4, and FT3/FT4 were 2.61, 1.35, and 60.61, respectively). Future studies can further investigate this area to enhance our understanding of its implications. Finally, as previously stated, the NHANES database provides concentrations of many nutrients but not iodine. Consequently, the classification of iodine nutritional status was based solely on urinary iodine concentration without incorporating dietary iodine intake data for a more comprehensive evaluation. Future investigations employing 24‐h dietary recalls or iodine‐specific food frequency questionnaires (FFQs) could unravel critical nuances currently obscured by sole reliance on urinary iodine concentrations. Such methods would quantify heterogeneity in iodine sources (e.g., dairy, seafood, iodized salt) and bioavailability, while concurrently revealing dose–response relationships between specific dietary iodine patterns and MAFLD severity. According to recent reports, iodine is expected to be included in the nutrient assessment panel of NHANES in future cycles. Prior to its official incorporation, dietary iodine intake can be estimated by combining iodine content data from selected food items provided by the U.S. Food and Drug Administration (FDA) with NHANES‐reported food consumption data [69]. Therefore, future studies should employ longitudinal designs and systematically collect both urinary and dietary iodine data to better elucidate the dynamic interplay between alterations in thyroid function and the progression of MAFLD across varying iodine nutritional statuses.

## 5. Conclusion

In conclusion, thyroid function indicators are associated with MAFLD, and this association is potentially mediated by ALT, HDL_C, and BMI. Moreover, the association between thyroid function indicators and MAFLD may potentially be modified by iodine nutritional status. Given the limitations of single urinary iodine measurements, future studies incorporating comprehensive dietary iodine assessments are needed to confirm these findings. Clinicians are advised to tailor the selection of thyroid function indicators based on patients’ iodine nutritional status to optimize the predictive accuracy for MAFLD. By integrating thyroid function screening and iodine nutritional status assessment, precise prevention and control of MAFLD can be achieved.

## Author Contributions

Wei Huang contributed to the conception and design. Wei Huang, Jiang Yao, and Heyong Huang contributed to the collection and assembly of data. Jiang Yao and Yunzhen Shi analyzed and interpreted the data.

## Funding

No funding was received for this research.

## Disclosure

All authors wrote and approved the final manuscript.

## Ethics Statement

In accordance with the Declaration of Helsinki, the Ethics Committee of Changxing People’s Hospital deemed that this research was based on open‐source data, so the need for ethics approval was waived.

## Consent

The authors have nothing to report.

## Conflicts of Interest

The authors declare no conflicts of interest.

## Supporting Information

Additional supporting information can be found online in the Supporting Information section.

## Supporting information


**Supporting Information** Table S1 Multicollinearity analysis between variables.

## Data Availability

The data that support the findings of this study are available from the corresponding author upon reasonable request.
