# Supplementary material for: Association Between Thyroid Function Indicators and Metabolic‐Associated Fatty Liver Disease: Effect Modification by Iodine Nutritional Status
Source: Int J Endocrinol. 2026 Jun 20;2026:9291433. doi: 10.1155/ije/9291433 (PMC13282677; doi:10.1155/ije/9291433)
Supplement: Supplementary file 1 — Supporting Information Table S1 Multicollinearity analysis between variables. [file IJE-2026-9291433-s001.docx]

**Table S1** Multicollinearity analysis between variables

| Model 1 | | Model 2 | |
| --- | --- | --- | --- |
| Variables | VIF | Variables | VIF |
| Age | 2 | Age | 1.812 |
| Race | 1.171 | Race | 1.160 |
| Education | 1.382 | Education | 1.374 |
| PIR | 1.372 | PIR | 1.358 |
| BMI | 7.413 | BMI | 1.552 |
| Waist | 8.035 |  |  |
| Drinking | 1.224 | Drinking | 1.199 |
| Smoking | 1.258 | Smoking | 1.242 |
| Iodine nutritional status | 1.072 | Iodine nutritional status | 1.070 |
| FPG | 3.168 |  |  |
| 2h-OGTT | 2.348 | 2h-OGTT | 1.959 |
| HbA1c | 2.804 | HbA1c | 1.891 |
| SBP | 1.657 | SBP | 1.672 |
| DBP | 1.311 | DBP | 1.295 |
| Albumin | 1.343 | Albumin | 1.353 |
| ALT | 2.254 | ALT | 2.237 |
| AST | 2.585 | AST | 2.564 |
| ALP | 1.187 | ALP | 1.181 |
| GGT | 1.898 | GGT | 1.890 |
| Uric acid | 1.468 | Uric acid | 1.422 |
| Platelet | 1.133 | Platelet | 1.131 |
| TG | 2075.734 | TG | 1.499 |
| LDL_C | 14310.266 | LDL_C | 1.135 |
| HDL_C | 3429.073 | HDL_C | 1.547 |
| TC | 19160.381 |  |  |

Model 1 included all the variables with inter-group differences. Model 2 excluded waist, FPG and TC. Abbreviations: VIF, variance inflation factor; PIR, poverty income ratio; BMI, body mass index; FPG, fasting plasma glucose; 2h-OGTT, 2-h post-load plasma glucose; HbA1c, hemoglobin A1c; SBP, systolic blood pressure; DBP, diastolic blood pressure; ALT, alanine aminotransferase; AST, aspartate aminotransferase; ALP, alkaline phosphatase; GGT, gamma glutamyl transferase; TG, triglyceride; LDL_C, low-density lipoprotein cholesterol; HDL_C, high-density lipoprotein cholesterol; TC, total cholesterol.
